# Differences in malaria vector biting behavior and changing vulnerability to malaria transmission in contrasting ecosystems of western Kenya

**DOI:** 10.1186/s13071-023-05944-5

**Published:** 2023-10-21

**Authors:** Irene Nzioki, Maxwell G. Machani, Shirley A. Onyango, Kevin K. Kabui, Andrew K. Githeko, Eric Ochomo, Guiyun Yan, Yaw A. Afrane

**Affiliations:** 1 Centre for Global Health Research, Kenya Medical Research Institute, Kisumu, Kenya; 2 School of Zoological Sciences, Kenyatta University, Nairobi, Kenya; 3 Program in Public Health, College of Health Sciences, University of California, Irvine, CA 92697 USA; 4 Department of Medical Microbiology, University of Ghana Medical School, College of Health Sciences, University of Ghana, Accra, Ghana

**Keywords:** Malaria vectors, Biting behavior, Human behavior, Western Kenya

## Abstract

**Background:**

Designing, implementing, and upscaling of effective malaria vector control strategies necessitates an understanding of when and where transmission occurs. This study assessed the biting patterns of potentially infectious malaria vectors at various hours, locations, and associated human behaviors in different ecological settings in western Kenya.

**Methods:**

Hourly indoor and outdoor catches of human-biting mosquitoes were sampled from 19:00 to 07:00 for four consecutive nights in four houses per village. The human behavior study was conducted via questionnaire surveys and observations. Species within the *Anopheles gambiae* complex and *Anopheles funestus* group were distinguished by polymerase chain reaction (PCR) and the presence of *Plasmodium falciparum* circumsporozoite proteins (CSP) determined by enzyme-linked immunosorbent assay (ELISA).

**Results:**

Altogether, 2037 adult female anophelines were collected comprising the *An. funestus* group (76.7%), *An. gambiae* sensu lato (22.8%), and *Anopheles coustani* (0.5%). PCR results revealed that *Anopheles arabiensis* constituted 80.5% and 79% of the *An. gambiae* s.l. samples analyzed from the lowland sites (Ahero and Kisian, respectively). *Anopheles gambiae* sensu stricto (hereafter *An. gambiae*) (98.1%) was the dominant species in the highland site (Kimaeti). All the *An. funestus* s.l. analyzed belonged to *An. funestus* s.s. (hereafter *An. funestus*). Indoor biting densities of *An. gambiae* s.l. and *An. funestus* exceeded the outdoor biting densities in all sites. The peak biting occurred in early morning between 04:30 and 06:30 in the lowlands for *An. funestus* both indoors and outdoors. In the highlands, the peak biting of *An. gambiae* occurred between 01:00 and 02:00 indoors. Over 50% of the study population stayed outdoors from 18:00 to 22:00 and woke up at 05:00, coinciding with the times when the highest numbers of vectors were collected. The sporozoite rate was higher in vectors collected outdoors, with *An. funestus* being the main malaria vector in the lowlands and *An. gambiae* in the highlands.

**Conclusion:**

This study shows heterogeneity of anopheline distribution, high outdoor malaria transmission, and early morning peak biting activity of *An. funestus* when humans are not protected by bednets in the lowland sites. Additional vector control efforts targeting the behaviors of these vectors, such as the use of non-pyrethroids for indoor residual spraying and spatial repellents outdoors, are needed.

**Graphical Abstract:**

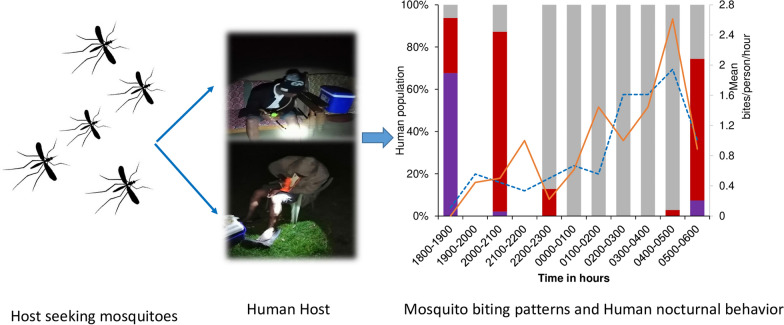

## Background

The wide-scale implementation of long-lasting insecticidal nets (LLINs) and indoor residual spraying (IRS) as vector control tools has led to a substantial decline in the malaria burden in sub-Saharan Africa in recent years [1]. However, progress has stalled since 2015, with the majority of African countries including Kenya experiencing persisting malaria transmission even with universal LLIN use and limited IRS deployment [1, 2]. It appears that a variety of factors are impeding the expected decrease in the incidence of malaria, for instance, widespread and increasing resistance to insecticides and drugs [3, 4], weak health systems, socioeconomic challenges, ecological changes, and climate change [5, 6]. Additionally, malaria vectors have shifted their behaviors to reduce exposure to insecticides [7, 8]. Such changes in vector populations threaten progress toward malaria elimination targets [9, 10]. Extensive investigations have been conducted on vector responses to control tools [3, 11, 12]; however, under the current vector control conditions, detailed studies are needed to understand the prevailing nocturnal human activities and vector biting behavior dynamics.

Indoor interventions rely on vector nocturnal human biting behavior. Historically, the primary malaria vectors *Anopheles gambiae* and *Anopheles funestus* have fed entirely indoors, with late-night peak biting activity [13]. This behavior coincides with the time most people are indoors and asleep. However, following the upscaling of control tools in sub-Saharan Africa, there is growing evidence of malaria vectors shifting their biting behaviors toward times and places where people are not protected [14–19]. Host choice and resting patterns have also been observed to change to evade insecticide-treated nets (ITNs) [15]. In Kenya, the National Malaria Control Program rolled out the universal bednet program in 2011, which led to an increase in the proportion of households owning at least one ITN, resulting in an increase in coverage from 56 to 80% in 2015 [20, 21]. Studies following the ITN universal coverage and IRS initiative have reported a shift in biting times [12, 18, 21–23] and biting locations [24] of the primary malaria vectors (*An. gambiae* and *An. funestus*) from the classical known behaviors. The majority of these studies have focused on vector behavior, with less or no attention to human habits and sleeping patterns in different eco-epidemiological settings.

The use of LLINs represents a powerful barrier against indoor biting and resting malaria vectors, but their efficacy is limited when people are not in bed, such as early morning or outdoors in the evening [25, 26]. Outdoor activities like farming and security jobs, as well as cultural practices, also increase the risk of malaria transmission, as they involve unprotected individuals overlapping with vector biting activity [25]. To achieve elimination, understanding local changes in vector biting behavior and identifying when and where people are exposed to vectors is crucial. This knowledge is key when evaluating the likely success of the current indoor mosquito control strategies and in designing effective interventions considering the local eco-epidemiological context. This study, therefore, assessed indoor and outdoor vector biting behavior and human habits and sleeping patterns potentially contributing to persistent malaria transmission in western Kenya.

## Methods

### Study sites

**Fig. 1:**
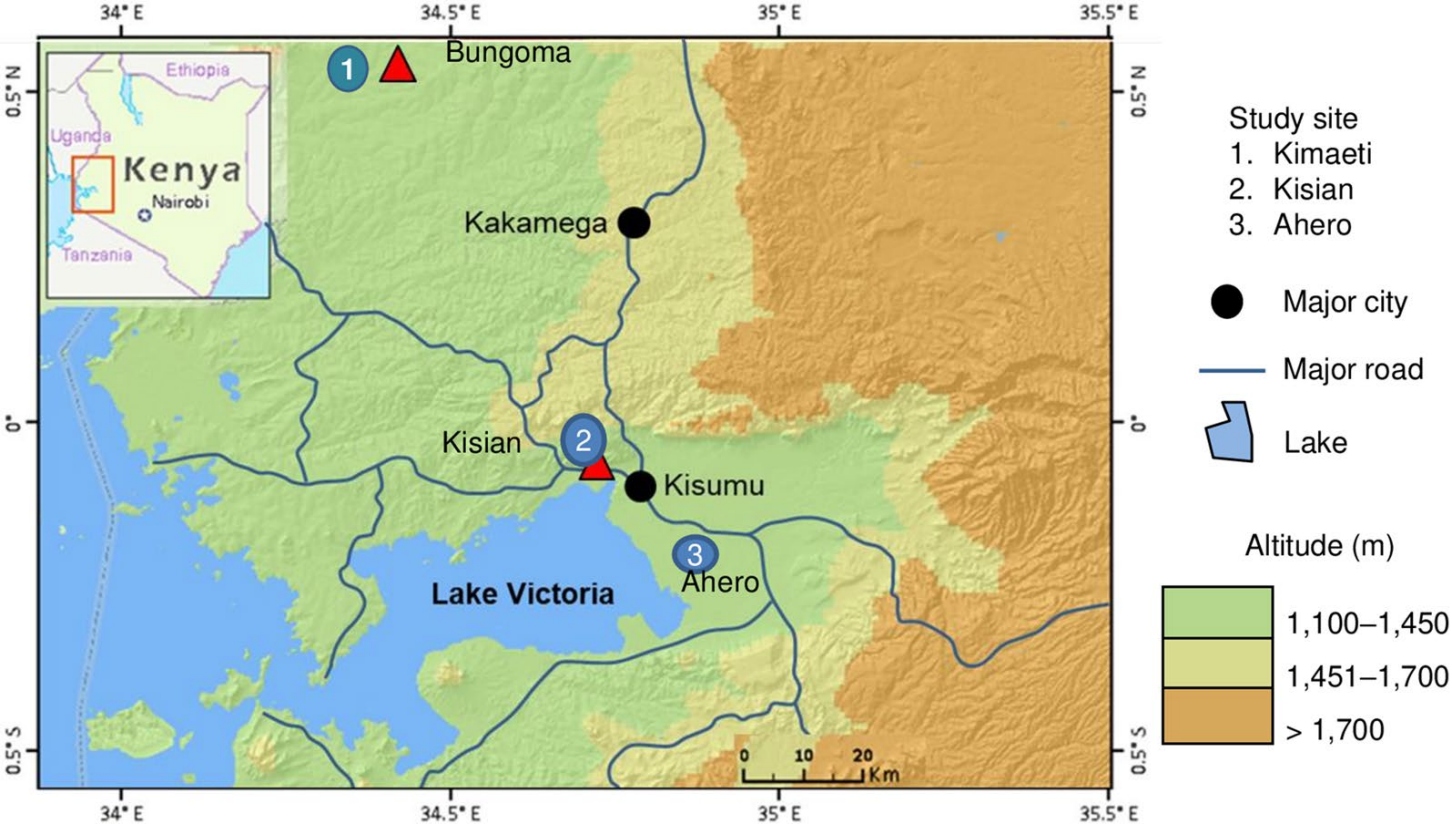
Map showing mosquito collection site in western Kenya

The study was carried out in Ahero (0° 0.11′ S, 34° 0.55′ E, altitude 1162 m above sea level), Kisian (00.0749° S, 034.6663° E, altitude 1137–1330 m above sea level), and Kimaeti (00.54057° N, 034.56410° E, altitude 1386–1545 m above sea level) (Fig. 1). The sites were selected based on past entomological studies [11, 21, 24, 27–29] and different environmental settings. The Ahero and Kisian sites are malaria-endemic areas in the lowland plains located adjacent to Lake Victoria in Kisumu County. The three malaria vector species, namely *Anopheles arabiensis, An. gambiae*, and *An. funestus*, are present, with *An. arabiensis* being the dominant species in the two lowland sites [21, 24]. Ahero is characterized by large irrigation (rice) fields and cattle farming, with the irrigation fields and frequent flooding providing favorable larval sites for malaria vector proliferation. Kisian is known for cattle farming, which provides vectors with alternative blood meal sources and brings them into increased contact with humans [28, 29]. Kimaeti is located in a malaria epidemic-prone highland area in Bungoma County, western Kenya. The area practices mixed farming, with the main cash crop being tobacco and cattle farming. The three malaria vectors are present in the highlands, with *An. gambiae* and *An. funestus* being the dominant species depending on the season [27, 30]. The highland and lowland sites of western Kenya have different levels of insecticide resistance [28].

The western Kenya region experiences a bimodal rainfall pattern, with the long rainy season from April to July, which is followed by increased malaria incidence and peak transmission. The short rainy season occurs from October to November. The hot and dry season is from January to March, which marks the lowest transmission period [2].

### Mosquito collection

Three weeks following the long rainy season in June–July 2021, mosquitoes were collected using the human landing catch (HLC) method in four fixed houses that were at least 300 m apart. Collections were conducted for four nights in each of the houses in all the study sites. Male adult volunteers, who acted as both bait and collector, were trained to minimize the variation between collectors and to avoid mosquito bites. A total of 16 volunteers were grouped into four teams. Each team consisted of four people, with two collecting mosquitoes indoors and the other two outside each house. The mosquitoes were captured when they attempted to bite a collector sitting on a chair exposing their lower legs. Collections were performed on four consecutive nights. The volunteers collected mosquitoes for 45 min, with a 15-min break, and changed their sitting position to avoid bias due to their attractiveness and skills. There were two collection shifts: one collector worked from 18:00 to 00:00 during each collection night, followed by the second collector from 00:00 to 07:00. A supervisor was assigned to coordinate the collection activity and carry out random spot checks during the collection nights to address any challenges and to keep the collectors awake. Participants were screened for malaria parasites and given anti-malaria prophylaxis drugs 1 week before the start of the study to avoid the risk of contracting malaria during the collection period. *Anopheles* mosquitoes collected each hour were identified morphologically the following morning using a dissecting microscope according to standard taxonomic keys described by Coetzee [31].

### Anopheline species molecular identification

The legs and wings of morphologically identified *An. gambiae* sensu lato and *An. funestus* s.l. specimens were used for DNA extraction using the ethanol precipitation method [32]. The sibling species of *An. gambiae* s.l. and *An. funestus* s.l. were distinguished using conventional polymerase chain reaction (PCR) [33, 34].

### Detection of sporozoites

Heads and thoraces of individual mosquitoes were used to test for the presence of *Plasmodium falciparum* sporozoites using enzyme-linked immunosorbent assay (ELISA) [35].

### Human behavior survey

A cross-sectional study design was employed to understand human activity and sleeping patterns in three villages. These behaviors were assessed during the same period that vector collections were carried out. Fifty households were randomly chosen and visited in each of the three villages studied. A household was defined as a house or a compound occupied by a group of individuals during the study. The inhabitants were interviewed using a questionnaire containing questions on where they slept at night, what time they slept at night and woke up in the morning, and the activities and cultural practices that kept them out at night. In cases where a household had more than one adult, individuals were interviewed separately to prevent them from influencing each other in their responses. In addition, data on bednet ownership (the proportion of households that owned at least one LLIN) and utilization (proportion of the population that had used LLINs the previous night) by the households and other intervention tools used for protection from mosquito bites were recorded. The start and end times of sleep periods and the time spent indoors and outdoors by inhabitants were determined from the data collected from the households.

### Data analysis

The human biting rates (HBRs), which indicate the density of *Anopheles*, were calculated by dividing the number of mosquitoes collected by the number of persons per night during the sampling period separately for indoor and outdoor venues [36]. Thus, throughout the study, total *Anopheles* density during the night and morning was evaluated as well as the hourly biting rate. The degree of indoor biting was calculated as indoor HBR 18:00 → 06:00/(indoor HBR 18:00 → 06:00 + outdoor HBR 18:00 → 06:00), while outdoor biting was calculated as outdoor HBR 18:00 → 06:00/(outdoor HBR 18:00 → 06:00 + indoor HBR 18:00 → 06:00) [37, 38]. The nocturnal biting activity of each *Anopheles* species was expressed as mean number of each *Anopheles* species landing per person per hour indoors or outdoors. The number of mosquitoes caught each hour is assumed to represent the number of mosquitoes attempting to feed on humans for the same period. The sporozoite rate was estimated as the proportion of mosquitoes positive for *P. falciparum* circumsporozoite protein (CSP) over the total number of mosquitoes tested. Descriptive statistics were used to summarize both household survey data and vector behavior data. The Chi-square test was also used to compare the indoor and outdoor biting rhythm of mosquitoes. The non-parametric Kruskal–Wallis rank-sum analysis was used to test for variation in biting rates among villages. In all analyses, *P* < 0.05 was considered significant. Data analysis was performed using the open-source R programming language software platform [39].

## Results

### Anopheline mosquito species composition and abundance

Overall, 2037 *Anopheles* females were collected from the three sites during the study period. Of these, 76.7% (*n* = 1565) were members of the *An. funestus* group, 22.8% (*n* = 465) belonged to *An. gambiae* s.l., and the remaining 0.5% (*n* = 7) belonged to the *An. coustani* group (Table 1). The *An. funestus* group was most abundant in Ahero, at 96.7% (*n* = 1570), followed by *An. gambiae* s.l. at 3% (*n* = 45) and *An. coustani* at 0.5% (*n* = 7). Out of 351 *Anopheles* females collected in Kisian, 86.6% (*n* = 304) were *An. gambiae* s.l. and 13.4% (*n* = 47) were *An. funestus* group species. In Kimaeti village, all the mosquitoes collected were *An. gambiae* s.l. (*n* = 116). Overall, 58.8% (95% CI 57–61%) of the mosquitoes were captured indoors and 41.2% (95% CI 39–43%) outdoors. The variation between the indoor and outdoor numbers of biting mosquitoes was statistically significant (*χ*^2^ = 121.7, *df* = 1, *P* < 0.0001).

**Table 1:** Summary of *Anopheles* species collected indoors and outdoors at different times of the night in Ahero, Kisian and Kimaeti villages

Site	*Anopheles* species	Indoor				Outdoor				Total overall
		Early night (18–22 h)	Night (00–03 h)	Early morning (03–07 h)	Total indoor	Early night (18–22 h)	Night (00–03 h)	Early morning (03–7 h)	Total outdoor	
Ahero	*An. funestus* s.l.	104	394	443	941	41	204	332	577	1518
	*An. gambiae* s.l.	7	8	7	22	9	2	12	23	45
	*An. coustani*	1	0	1	2	1	0	4	5	7
	Overall	112	402	451	965	51	206	348	605	1570
Kisian	*An. funestus* s.l.	0	14	23	37	0	2	8	10	47
	*An. gambiae* s.l.	35	37	59	131	39	53	81	173	304
	Overall	35	51	82	168	39	55	89	183	351
Kimaeti	*An. gambiae* s.l.	19	29	19	67	12	21	16	49	116
	Total	166	482	552	1200	102	282	453	837	2037

Molecular identification confirmed all *An*. *funestus* s.l. assayed from the Ahero and Kisian sites to be *An*. *funestus*. *Anopheles arabiensis* was most abundant in the lowland sites [Ahero 80.5% (95% CI 68.4–92.6%), Kisian 79% (95% CI 75.2–85.1%)], followed by *An. gambiae* [19.3% (95% CI 7.4–31.6%) and 21% (95% CI 14.8–24.8%), respectively]. In Kimaeti, *An. gambiae* [98.1% (95% CI 95.5–100%)] was the dominant species, followed by *An. arabiensis* [2% (95% CI 0.7–4.5%)]. The relative proportion of *Anopheles* species in mosquito samples varied significantly among the sites (*χ*^2^ = 22.9, *df* = 2, *P* < 0.001).

### Hourly biting patterns of anophelines

**Fig. 2:**
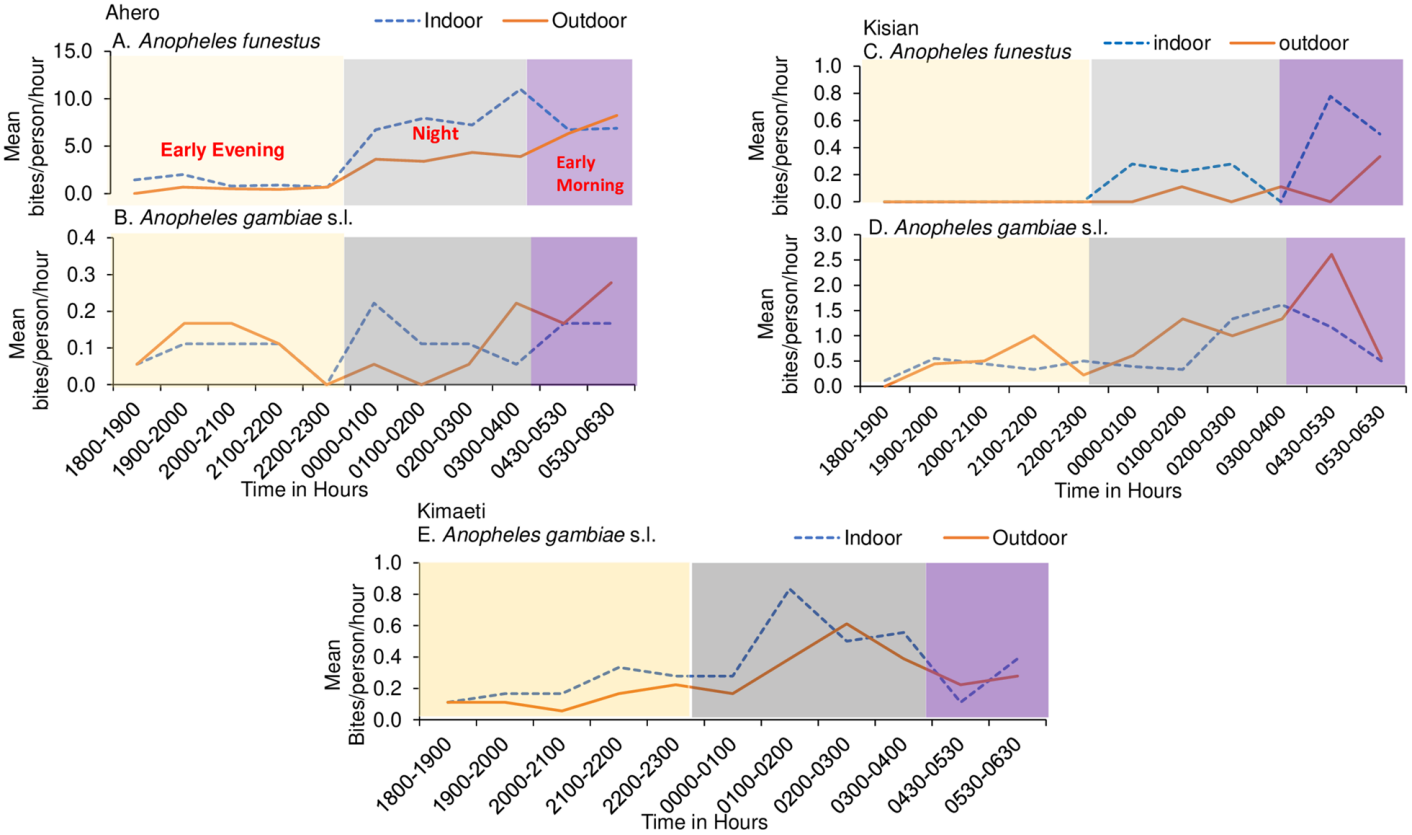
Mean hourly human biting patterns of the *Anopheles* species in **A**, **B** Ahero (*An. funestus* and *An. gambiae* s.l.), **C**, **D** Kisian (*An. funestus* and *An. gambiae* s.l.), and **E** Kimaeti (*An. gambiae* s.l.)

The human biting activity of *An. funestus* in Ahero was observed from dawn to dusk both indoors and outdoors, with gradual peaks from midnight (00:00 to 01:00) (mean 7.9 bites/person/hour) and a maximum peak at dawn (03:00–04:00) (mean 11.0 bites/person/hour) indoors. *Anopheles funestus* showed a steady increase in the late morning, with peak biting activity at 05:30–06:30 (8.2 mean bites/person/hour) outdoors (Fig. 2A) when people were out of bed. The biting activity of *An. arabiensis* was generally higher outdoors than indoors, with two peaks indoors at midnight and another one late morning, 05:00–06:30 (mean 0.2 bites/person/hour). Increased outdoor biting activity was observed in the early evening between 19:00 and 20:00, and was pronounced in the late morning between 05:30 and 06:30 (0.3 bites/person/hour; Fig. 2B).

On the other hand, *An. funestus* in Kisian showed a steady increase in late morning activity, with peak biting activity at 04:30–05:30 (mean, 0.8 bites/person/hour) indoors. The outdoor peak biting activity began at 04:30–06:30 (mean, 0.4 bites/person/hour; Fig. 2C). The biting activity of *An. gambiae* s.l. was pronounced at the end of midnight indoors (mean, 1.6 bites/person/hour, Fig. 2D). The outdoor biting activity was bimodal, with an early and smaller peak at 21:00–22:00 and a major peak late morning at 04:30–05:30 (mean, 2.6 bites/person/hour; Fig. 2D), with activity then declining progressively during the morning.

**Fig. 3:**
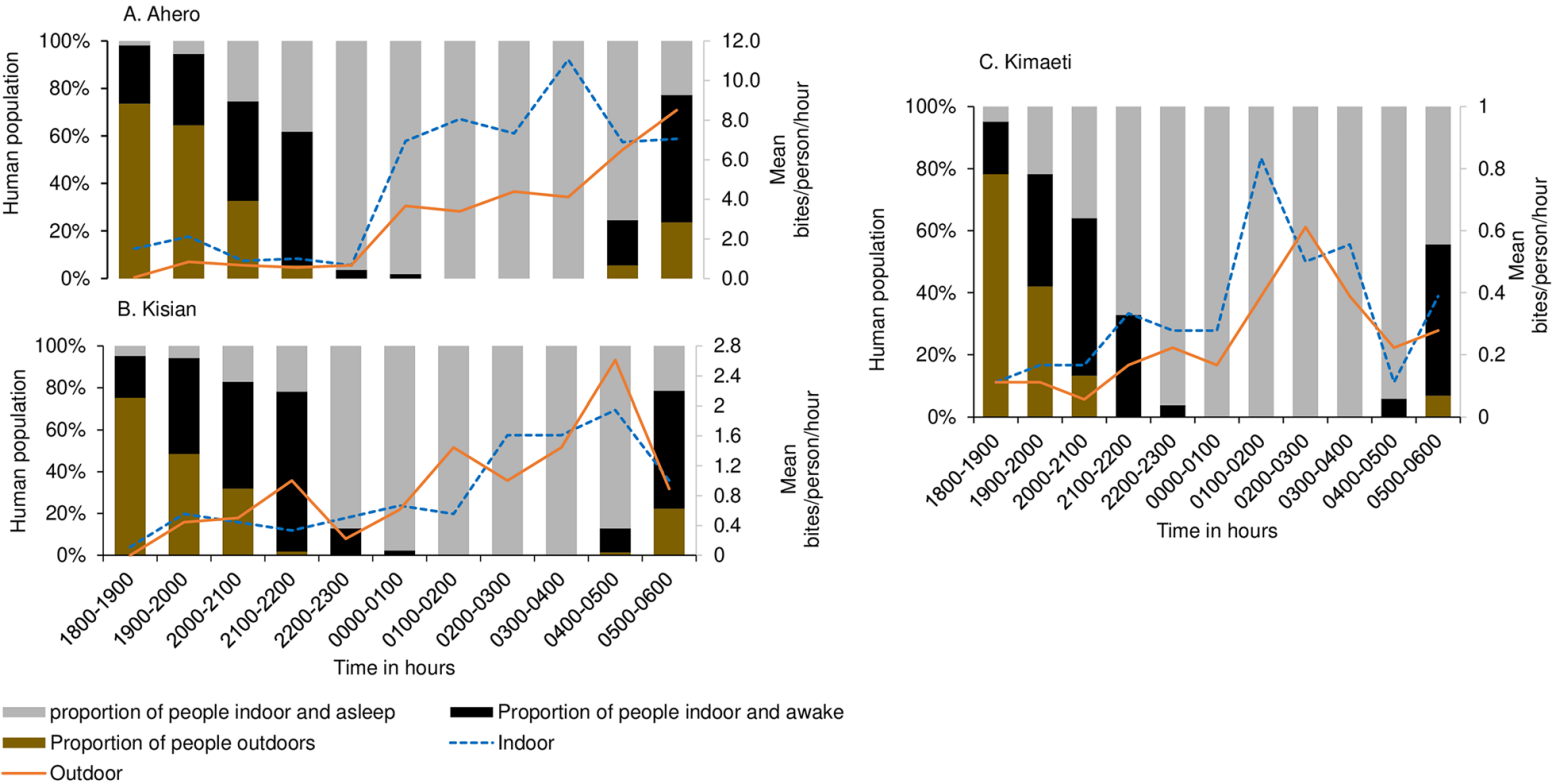
Indoor and outdoor mean hourly biting rates of *Anopheles* mosquitoes with the proportion of people outdoors, indoors and awake, and indoors and asleep throughout the night in **A** Ahero, **B** Kisian, and **C** Kimaeti

In the highlands (Kimaeti), the biting activity of *An. gambiae* s.l. (mostly *An. gambiae* sensu stricto) indoors was bimodal, with a major peak at midnight, 01:00–02:00 (mean, 0.8 bites/person/hour; Fig. 2E), when people were asleep and another one early in the morning, 03:00–04:00 (mean, 0.6 bites/person/hour; Fig. 2E), when people were likely to be awake. The outdoor activity peaked late at midnight from 02:00 to 03:00. Additional information regarding biting based on the proportion of people indoors/outdoors and asleep/awake is given in Fig. 3.

### Anopheline indoor and outdoor biting activity

Overall, the majority of *An. funestus* collected from Ahero and Kisian exhibited endophagic behavior (preference for feeding indoors) (Ahero, 62% and Kisian, 78.7%; Table 2), while *An. gambiae* s.l. (mostly *An. arabiensis*) preferred feeding outdoors (exophagy) (Ahero, 51.1% and Kisian, 56.7%, respectively; Table 2). Low numbers of *An. coustani* were collected in Ahero (*n* = 7), and the majority were collected outdoors (5/7). The indoor HBR of *An. funestus* was higher than outdoor HBR in Ahero [52.3 vs. 32.1 mosquitoes/person/night (m/p/n)] and Kisian (2.1 vs. 0.5 m/p/n, respectively). The HBR for *An. arabiensis* was slightly higher outdoors than indoors in Kisian (10 vs. 7 m/p/n), while in Ahero it was similar between indoors and outdoors (1.2 m/p/n).

**Table 2:** Feeding behaviors of *Anopheles* species collected in Ahero, Kisian, and Kimaeti villages in western Kenya

Site	*Anopheles* species	Biting activity			
		Indoor (%)	Outdoor (%)	Protected hours (%)	Unprotected hours (%)
Ahero	*An. gambiae* s.l.	48.9	51.1	33.3	66.7
	*An. funestus* s.l.	62.0	38.0	57.0	43.0
	*An. gambiae* s.s.	66.7	33.3	51.5	48.5
	*An. arabiensis*	31.4	68.6	30.0	62.5
	*An. funestus* s.s.	60.4	39.6	58.9	41.8
Kisian	*An. gambiae* s.l.	43.1	56.9	47.0	53.0
	*An. funestus* s.l.	78.7	21.3	38.3	61.7
	*An. gambiae* s.s.	50.3	29.6	59.3	40.4
	*An. Arabiensis*	86.1	23.8	22.2	69.4
	*An. funestus* s.s.	80.0	13.0	66.7	28.6
Kimaeti	*An. gambiae* s.l.	57.8	42.2	33.6	66.4
	*An. gambiae* s.s.	62.5	37.5	41.5	58.7

In the highland site (Kimaeti), 57.8% of *An. gambiae* s.l. (mostly *An. gambiae* s.s.) collected were indoors, clearly indicating the preference of this species for feeding indoors (endophagy). The indoor HBR for *An. gambiae* s.l. was 3.7 m/p/n and outdoors was 2.7 m/p/n.

### Sporozoite infectivity rates

In total, 489 *An. funestus*, 337 *An. gambiae*, 51 *An. arabiensis*, and seven *An. coustani* samples were tested for the presence of *P. falciparum* CSP. Overall, four samples (two Ahero and two Kimaeti) tested positive, yielding an infection rate of 0.4% (2/474) in Ahero and 1.9% (2/105) in Kimaeti. In Ahero, only *An. funestus* mosquitoes collected indoors (0.3%) and outdoors (0.5%) were positive for *P. falciparum* CSP (Table 3). In Kimaeti, CSP was detected in the indoor and outdoor *An. gambiae* collections, with infectivity rates of 1.5% and 2.6%, respectively. No CSP positivity was detected in *An. arabiensis* or *An. coustani* samples assayed or for mosquitos collected from Kisian (*n* = 244).

**Table 3:** Indoor and outdoor sporozoite rates of *Anopheles* mosquitoes collected from Ahero, Kisian, and Kimaeti villages in western Kenya

Study site	Sibling species	No. tested	Indoor (%)	No. tested	Outdoor (%)	Overall (Pf+)
Sporozoite infection rate						
Ahero	*An. gambiae* s.s.	21	0	12	0	0
	*An. arabiensis*	6	0	13	0	0
	*An. funestus* s.s.	291	1 (0.3)	183	1 (0.5)	2 (0.4)
	*An. Coustani*	2	0	5	0	0
Kisian	*An. gambiae* s.s.	100	0	99	0	0
	*An. arabiensis*	25	0	5	0	0
	*An. funestus* s.s.	12	0	3	0	0
Kimaeti	*An. gambiae* s.s.	66	1 (1.5)	39	1 (2.6)	2 (1.9)
	*An. arabiensis*	2	0	0	0	0

Pf+ = *P. falciparum* positivity

### Human exposure to mosquito bites and protection by LLINs

The survey showed that LLIN use was high across the three study sites, with 91%, 99%, and 96.6% of households in Kisian, Ahero, and Kimaeti, respectively, having at least one LLIN. LLINs were the primary prevention method against mosquito bites and malaria infection. Overall, over 50% of the study participants reported having stayed outdoors or outdoors and indoors until 21:00 (Fig. 3). About 77% of the respondents reported going to bed by 22:00. In Ahero, 54% of preschool-age children had gone to sleep, and 35% of school-going children, 86% of adult women in Ahero, 46% in Kimaeti, and none in Kisian had gone to sleep by 22:00, while 14% of men were asleep by 22:00. Overall, at 23:00, the majority (93%) of the respondents were asleep while 7% were awake indoors.

Across the study sites, it was observed that waking time was between 04:00 and 07:00. About 10% of respondents were awake but indoors in the early morning (04:00) in Kisian and Ahero, coinciding with the time of high mosquito biting (Fig. 3A, B). At 05:00, about 60% were awake and outdoors across the three sites, and nearly all exposure to malaria vectors peaked at this time (Fig. 3).

**Fig. 4:**
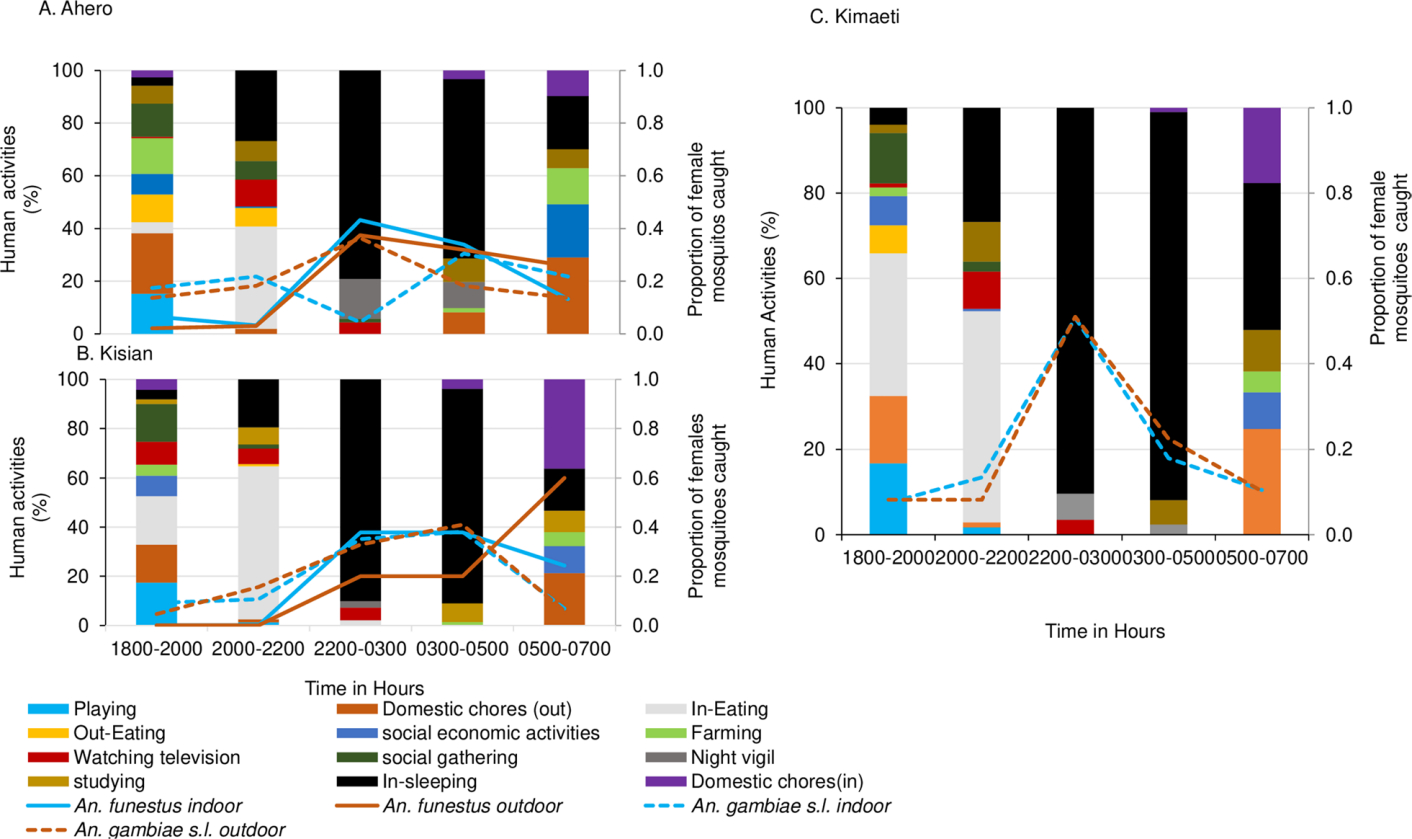
Indoor- and outdoor-specific human activities overlaid with the proportion of mosquitoes caught throughout the night in **A** Ahero, **B** Kisian, and **C** Kimaeti

The main activities that kept people outdoors between 18:00 and 20:00 included household (domestic) chores, playing, social–economic activities (i.e., selling at grocery stores), and social gatherings (Fig. 4). Night vigils and watching television after dinner were reported to keep the majority of men awake longer than their female counterparts. Respondents woke early in the morning—for instance, women to prepare breakfast and children to go to school, and milking and other domestic chores including cleaning their houses and livestock areas. Agricultural activities were also a major reason that people woke up early in the morning, in particular in Ahero, where rice plantation is the main activity.

## Discussion

Understanding the biting behavior of malaria vectors, including the period, location. and frequency at which humans are exposed to infectious mosquito bites in the field, plays a crucial role in the fight against malaria. This study outlines the variety of *Anopheles* nocturnal biting activity in different eco-epidemiological settings (highland and lowland areas of western Kenya), with data on human behavior that influence when and where disease transmission may occur. Overall, *An. funestus*, *An. gambiae* s.l., and *An. coustani* were found to be the three human-biting *Anopheles* species occurring both indoors and outdoors. *Anopheles funestus* and *An. gambiae* were the dominant vectors biting humans indoors, while *An. arabiensis* and *An. coustani* were more likely to bite outdoors. The study further revealed early evening and late morning biting behavior both indoors (when people are still active and unprotected by LLINs) and outdoors. These behaviors have implications for the risk of malaria transmission and the effectiveness of interventions, particularly those that target human-feeding vectors indoors.

The study revealed *An. funestus* as the predominant vector biting humans in Ahero, while *An. gambiae* s.l. prevailed in Kisian and Kimaeti. This difference in species abundance was attributed to the type of breeding habitats available in the study sites, season, degree of predisposition to biting humans, scaling up of insecticide-based interventions [40–43], and mosquito sampling method employed [40, 44]. For instance, *An. funestus* is known to breed in permanent habitats with aquatic vegetation cover [41], habitats typically found in Ahero rice irrigation schemes. *Anopheles gambiae* and *An. arabiensis* prefer breeding in small, sunlit temporary water pools [45], the type of habitats found in the Kisian and Kimaeti areas [46]. Studies on the distribution of anopheline species in rice fields have documented a succession between *An. arabiensis* and *An. funestus* [40, 47]. The increased abundance of *An. funestus* indicates a significant contribution of this species in the transmission of malaria in this region despite the widespread use of LLINs.

*Anopheles funestus* and *An. gambiae* exhibited endophagic behavior, with higher proportions seeking a host indoors than outdoors. These findings corroborate earlier reports from western Kenya documenting a greater likelihood of host-seeking indoors than outdoors for the two primary vectors [18, 21, 24]. In Ahero (lowland site), as expected, *An. arabiensis* preferred to bite outdoors. In contrast, a higher proportion of *An. arabiensis* were caught biting indoors in Kisian (lowland site), demonstrating that mosquito foraging behavior can vary noticeably within relatively small areas. The outdoor biting activity of this species in Ahero was found to be largely associated with cattle availability in the region, although this was not quantified in this study. Recent studies in Kisian have noted increased levels of insecticide resistance in *An. arabiensis* caught resting indoors versus outdoors [28], and this could also explain the observed variations in biting activity. Of concern is the fraction of *An. gambiae* and *An. funestus* observed biting outside the classical time (midnight) and whether these behaviors represent resilience or resistance, as this appears to reduce their chance of encountering indoor interventions (IRS and LLINs) [15, 48]. The secondary vector *An. coustani* was found to prefer foraging outdoors in Ahero (albeit in very low numbers, *n* = 7). Although this vector is not given much attention due to its exophagic and zoophilic feeding preferences [40], it has been reported to be susceptible to *P. falciparum* infections [24, 49, 50]. The outdoor human biting activity observed in the current study also implies that it has a potential role in malaria transmission, pointing to the need for integrated vector management (IVM) control strategies with a combination of non-chemical and chemical methods for more effective vector management—for instance, biological larval source management, attractive toxic sugar baits (ATSBs), and spatial repellents.

The biting behaviors of *An. arabiensis* in the lowland sites revealed an early peak in the evening (19:00–20:00) outdoors and intense biting activity late in the morning (04:30–06:30) both indoors and outdoors, a time when local people are awake and not protected by LLINs. Our findings are in agreement with previous studies from the same regions [18, 40] and elsewhere in Africa [26, 51] that observed an increase in morning outdoor biting of this species between 03:00 and 05:00. The increased biting activity outdoors despite the equal chances of females biting the human bait in either of the two locations (indoors vs. outdoors) may have arisen from its preference for host-seeking outdoors. *Anopheles arabiensis* is known to be flexible in behaviors, and in the presence of LLINs indoors and livestock outdoors, human–vector contact is likely to be minimal as the vector seeks an alternative host [24, 40, 52]. *Anopheles funestus* was responsible for most vectors biting indoors in the lowlands, and this observation accords with previous findings in the same region [21]. In contrast to early studies on biting behavior, which reported this vector maintaining its classical biting habits (late night) in the same regions [11, 12], this study revealed a shift from classical to late-morning biting activity (05:30–06:30) indoors and outdoors in both lowland sites. A plausible explanation for the extended periods of foraging to late in the morning could be a failure to access the preferred host (human) during the feeding hours (late night), forcing the mosquito to wait for the times the host is unprotected. Previous studies in western Kenya have shown pre-biting resting behavior in *An. funestus*, where the vectors were seen resting on the walls before attacking the host [40, 47]. Recent studies have reported shifts in the biting behavior of *An. funestus* after universal LLIN coverage and IRS, from its historical biting times (late night) to late morning or daytime biting [18, 22, 23]; however, it is not clear whether this behavior is due to plasticity or has a genetic basis. The observed behavior is worrisome, as this species (*An. funestus*) is efficient in malaria transmission [24, 53, 54], and biting during times that people are not protected (indoors and outdoors) presents a gap in protection.

In the highlands (Kimaeti), only *An. gambiae* were collected, with previous studies confirming the species to be dominant in the region [27, 28]. This vector showed no change in biting activity, as the results indicate that the species preferred feeding indoors, with pronounced activity late at night between 01:00 and 02:00. Historical studies have reported humans as the principal host for this species, unlike its sibling species *An. arabiensis* [13]. The persistence in feeding late at night indoors when people are likely to be protected by LLINs can be partially explained by increased resistance levels observed in this species [28, 55]. Machani et al. [56] investigated the host-seeking activity of highly pyrethroid-resistant *An. gambiae* when a human bait was protected with a treated LLIN, and observed that, unlike susceptible mosquitoes, resistant mosquitoes attempted to bite a host sleeping under a treated bednet. The late-night biting behavior indoors by *An. gambiae* implies that compliance with LLIN usage could offer protection from infective bites during this period, as the peaks correspond to the times of sleeping. Of concern is the small peak observed in the early morning indoors (03:00–04:00) when people are waking up and remain unprotected by LLINs, as this could have implications for persistence of malaria transmission indoors.

*Anopheles funestus* and *An. gambiae* were responsible for malaria transmission both indoors and outdoors in the lowland and highland sites, respectively, with the majority of malaria infections occurring outdoors. These findings are in agreement with previous studies that observed the two vectors to be the main drivers of malaria transmission in the region [21, 24, 27]; however, contrary to the present study, the earlier studies reported high infection rates indoors. It is worth mentioning that high bednet ownership and usage of > 90% was confirmed in all three sites. The reaction of malaria vectors to indoor-based interventions such as the excito-repellence effects of pyrethroids used in LLINs [22] may force mosquitoes to shift their biting times, thus explaining the increase in outdoor transmission. This phenomenon can be exacerbated by human behavior in areas where people remain outdoors for long periods without protection [57]. In this study, over 50% of the population interviewed stayed outdoors or between outdoors and indoors until 21:00. The majority of the respondents were asleep by 23:00 (93%), and the waking time across the sites was between 04:00 and 07:00, with about 10% waking up and staying indoors at 04:00 and about 60% observed outdoors in the morning at 05:00. Human behavior coincides with the vector biting patterns observed in this study. Previous reports indicated that people spend more time outdoors before retiring to bed [21], with a high risk of infectious bites from *An. funestus* outdoors. Agricultural practices (rice farming, milking), domestic chores, and social–economic activities (selling at grocery stalls) were the main activities that kept people outdoors. Elsewhere, electricity has been shown to influence community outdoor activity and sleeping times, as people stay up or out of bed for longer in the evening hours [57, 58], although in this study we did not record the number of houses with electricity. However, this can be confirmed in this study, as men were observed watching television indoors and going to social gatherings (to watch football games) for longer hours in the evening. Therefore, the study findings support previous claims that current control strategies focusing on indoor-based interventions may not be sufficient to eliminate malaria transmission in most endemic regions [59].

## Conclusion

*Anopheles funestus* and *An. gambiae* were found to be responsible for malaria transmission in the region. The shifting in time of biting from classical biting to late morning biting (indoor and outdoor) of *An. funestus* and the early evening outdoor biting of *An. arabiensis*, together with the high outdoor malaria transmission, could be due to pressure from the LLINs or humans spending more time unprotected outdoors. These findings have important implications for the epidemiology and strategies for the control of malaria in the study area. Additional control strategies are needed for ongoing interventions to better address the issue of residual transmission and reduce indoor and outdoor biting vectors using a more diverse toolbox with IVM strategies.

## Data Availability

The dataset supporting the conclusions of this article is included within the article.

## Abbreviations

HLC: Human landing catch
HBR: Human biting rate
CSP: Circumsporozoite protein
ITN: Insecticide-treated net
LLIN: Long-lasting insecticidal net
IRS: Indoor residual spraying

## References

[1] WHO, 2020. World malaria report 2020: 20 years of global progress and challenges.

[2] Zhou G, Afrane YA, Vardo-Zalik AM, Atieli H, Zhong D, Wamae P (2011). Changing patterns of malaria epidemiology between 2002 and 2010 in Western Kenya: the fall and rise of malaria. PLoS ONE.

[3] Ranson H, Lissenden N (2016). Insecticide resistance in African *Anopheles* mosquitoes: a worsening situation that needs urgent action to maintain malaria control. Trends Parasitol.

[4] WHO, 2022. World malaria report 2022.

[5] Okumu F, Gyapong M, Casamitjana N, Castro MC, Itoe MA, Okonofua F (2022). What Africa can do to accelerate and sustain progress against malaria. PLoS Glob Public Health.

[6] Yewhalaw D, Kweka EJ, Yewhalaw D, Kweka EJ. Insecticide resistance in East Africa—history, distribution and drawbacks on malaria vectors and disease control. Insecticides resistance; 2016.

[7] Killeen GF, Chitnis N (2014). Potential causes and consequences of behavioural resilience and resistance in malaria vector populations: a mathematical modelling analysis. Malar J.

[8] Carrasco D, Lefèvre T, Moiroux N, Pennetier C, Chandre F, Cohuet A (2019). Behavioural adaptations of mosquito vectors to insecticide control. Curr Opin Insect Sci.

[9] Antonio-Nkondjio C, Kerah CH, Simard F, Awono-Ambene P, Chouaibou M, Tchuinkam T (2006). Complexity of the malaria vectorial system in Cameroon: contribution of secondary vectors to malaria transmission. J Med Entomol.

[10] Killeen GF, Seyoum A, Sikaala C, Zomboko AS, Gimnig JE, Govella NJ (2013). Eliminating malaria vectors. Parasit Vectors.

[11] Githeko AK, Mbogo CM, Atieli FK (1996). Resting behaviour, ecology and genetics of malaria vectors in large-scale agricultural areas of Western Kenya. Parassitologia.

[12] Bayoh MN, Walker ED, Kosgei J, Ombok M, Olang GB, Githeko AK (2014). Persistently high estimates of late night, indoor exposure to malaria vectors despite high coverage of insecticide treated nets. Parasit Vectors.

[13] Gillies M, Coetzee M (1987). A supplement to the Anophelinae of Africa south of the Sahara (Afrotropical Region). Publ Sth Afr Inst Med Res.

[14] Briët OJ, Chitnis N (2013). Effects of changing mosquito host searching behaviour on the cost effectiveness of a mass distribution of long-lasting, insecticidal nets: a modelling study. Malar J.

[15] Gatton ML, Chitnis N, Churcher T, Donnelly MJ, Ghani AC, Godfray HCJ (2013). The importance of mosquito behavioural adaptations to malaria control in Africa. Evolution.

[16] Reddy MR, Overgaard HJ, Abaga S, Reddy VP, Caccone A, Kiszewski AE (2011). Outdoor host seeking behaviour of *Anopheles gambiae* mosquitoes following initiation of malaria vector control on Bioko Island. Equator Guinea Malar J.

[17] Cooke MK, Kahindi SC, Oriango RM, Owaga C, Ayoma E, Mabuka D (2015). ‘A bite before bed’: exposure to malaria vectors outside the times of net use in the highlands of western Kenya. Malar J.

[18] Wamae PM, Githeko AK, Otieno GO, Kabiru EW, Duombia SO (2015). Early biting of the *Anopheles gambiae* s.s. and its challenges to vector control using insecticide treated nets in western Kenya highlands. Acta Trop.

[19] Meyers JI, Pathikonda S, Popkin-Hall ZR, Medeiros MC, Fuseini G, Matias A (2016). Increasing outdoor host-seeking in *Anopheles gambiae* over 6 years of vector control on Bioko Island. Malar J.

[20] Zhou G, Li JS, Ototo EN, Atieli HE, Githeko AK, Yan G (2014). Evaluation of universal coverage of insecticide-treated nets in western Kenya: field surveys. Malar J.

[21] Ototo EN, Mbugi JP, Wanjala CL, Zhou G, Githeko AK, Yan G (2015). Surveillance of malaria vector population density and biting behaviour in Western Kenya. Malar J.

[22] Moiroux N, Gomez MB, Pennetier C, Elanga E, Djènontin A, Chandre F (2012). Changes in *Anopheles funestus* biting behavior following universal coverage of long-lasting insecticidal nets in Benin. J Infect Dis.

[23] Abong’o B, Gimnig JE, Torr SJ, Longman B, Omoke D, Muchoki M (2020). Impact of indoor residual spraying with pirimiphos-methyl (Actellic 300CS) on entomological indicators of transmission and malaria case burden in Migori County, Western Kenya. Sci Rep.

[24] Degefa T, Yewhalaw D, Zhou G, Lee M, Atieli H, Githeko AK (2017). Indoor and outdoor malaria vector surveillance in western Kenya: implications for better understanding of residual transmission. Malar J.

[25] Monroe A, Moore S, Okumu F, Kiware S, Lobo NF, Koenker H (2020). Methods and indicators for measuring patterns of human exposure to malaria vectors. Malar J.

[26] Doucoure S, Thiaw O, Wotodjo AN, Bouganali C, Diagne N, Parola P (2020). *Anopheles arabiensis* and *Anopheles funestus* biting patterns in Dielmo, an area of low level exposure to malaria vectors. Malar J.

[27] Machani MG, Ochomo E, Amimo F, Kosgei J, Munga S, Zhou G (2020). Resting behaviour of malaria vectors in highland and lowland sites of western Kenya: implication on malaria vector control measures. PLoS ONE.

[28] Owuor KO, Machani MG, Mukabana WR, Munga SO, Yan G, Ochomo E (2021). Insecticide resistance status of indoor and outdoor resting malaria vectors in a highland and lowland site in Western Kenya. PLoS ONE.

[29] Mustapha AM, Musembi S, Nyamache AK, Machani MG, Kosgei J, Wamuyu L (2021). Secondary malaria vectors in western Kenya include novel species with unexpectedly high densities and parasite infection rates. Parasit Vectors.

[30] Ndenga B, Githeko A, Omukunda E, Munyekenye G, Atieli H, Wamai P (2014). Population dynamics of malaria vectors in western Kenya highlands. J Med Entomol.

[31] Coetzee M (2020). Key to the females of Afrotropical *Anopheles* mosquitoes (Diptera: Culicidae). Malar J.

[32] Collins FH, Mendez MA, Rasmussen MO, Mehaffey PC, Besansky NJ, Finnerty V (1987). A ribosomal RNA gene probe differentiates member species of the *Anopheles gambiae* complex. Am J Trop Med Hyg.

[33] Scott JA, Brogdon WG, Collins FH (1993). Identification of single specimens of the *Anopheles gambiae* complex by the polymerase chain reaction. Am J Trop Med Hyg.

[34] Cohuet A, Simard F, Toto J-C, Kengne P, Coetzee M, Fontenille D (2003). Species identification within the *Anopheles funestus* group of malaria vectors in Cameroon and evidence for a new species. Am J Trop Med Hyg.

[35] Wirtz RA, Ballou WR, Schneider I, Chedid L, Gross MJ, Young JF (1987). *Plasmodium falciparum*: Immunogenicity of circumsporozoite protein constructs produced in *Escherichia coli*. Exp Parasitol.

[36] Kabbale FG, Akol AM, Kaddu JB, Onapa AW (2013). Biting patterns and seasonality of *Anopheles gambiae* sensu lato and *Anopheles funestus* mosquitoes in Kamuli District, Uganda. Parasit Vectors.

[37] Williams J, Pinto J. Training manual on malaria entomology for entomology and vector control technicians (basic level). USAID Washington, DC. 2012;78.

[38] Kenea O, Balkew M, Tekie H, Gebre-Michael T, Deressa W, Loha E (2016). Human-biting activities of *Anopheles* species in south-central Ethiopia. Parasit Vectors.

[39] Team R. Core development team, R: R Foundation for Statistical Computing. Computing, ed, Vienna, Austria; 2016.

[40] Githeko AK, Service MW, Mbogo CM, Atieli FA, Juma FO (1994). Sampling *Anopheles arabiensis*, *Anopheles gambiae* sensu lato and *Anopheles funestus* (Diptera: Culicidae) with CDC light traps near a rice irrigation area and a sugarcane belt in western Kenya. Bull Entomol Res.

[41] Minakawa N, Munga S, Atieli F, Mushinzimana E, Zhou G, Githeko AK (2005). Spatial distribution of anopheline larval habitats in Western Kenyan highlands: effects of land cover types and topography. Am J Trop Med Hyg.

[42] Mwangangi JM, Mbogo CM, Orindi BO, Muturi EJ, Midega JT, Nzovu J (2013). Shifts in malaria vector species composition and transmission dynamics along the Kenyan coast over the past 20 years. Malar J.

[43] McCann RS, Ochomo E, Bayoh MN, Vulule JM, Hamel MJ, Gimnig JE (2014). Reemergence of *Anopheles funestus* as a vector of *Plasmodium falciparum* in Western Kenya after long-term implementation of insecticide-treated bed nets. Am J Trop Med Hyg.

[44] Mathenge EM, Misiani GO, Oulo DO, Irungu LW, Ndegwa PN, Smith TA (2005). Comparative performance of the Mbita trap, CDC light trap and the human landing catch in the sampling of *Anopheles arabiensis*, *An. funestus* and culicine species in a rice irrigation in western Kenya. Malar J.

[45] Gimnig JE, Ombok M, Kamau L, Hawley WA (2001). Characteristics of larval anopheline (Diptera: Culicidae) habitats in Western Kenya. J Med Entomol.

[46] Debrah I, Afrane YA, Amoah LE, Ochwedo KO, Mukabana WR, Zhong D (2021). Larval ecology and bionomics of *Anopheles funestus* in highland and lowland sites in western Kenya. PLoS ONE.

[47] Chandler JA, Highton RB, Hill MN (1975). Mosquitoes of the Kano Plain, Kenya. I. Results of indoor collections in irrigated and non-irrigated areas using human bait and light traps. J Med Entomol.

[48] Russell TL, Govella NJ, Azizi S, Drakeley CJ, Kachur SP, Killeen GF (2011). Increased proportions of outdoor feeding among residual malaria vector populations following increased use of insecticide-treated nets in rural Tanzania. Malar J.

[49] Mwangangi JM, Muturi EJ, Muriu SM, Nzovu J, Midega JT, Mbogo C (2013). The role of *Anopheles arabiensis* and *Anopheles coustani* in indoor and outdoor malaria transmission in Taveta District. Kenya Parasit Vectors.

[50] Goupeyou-Youmsi J, Rakotondranaivo T, Puchot N, Peterson I, Girod R, Vigan-Womas I (2020). Differential contribution of *Anopheles coustani* and *Anopheles arabiensis* to the transmission of *Plasmodium falciparum* and *Plasmodium vivax* in two neighbouring villages of Madagascar. Parasit Vectors.

[51] Kibret S, Wilson G (2016). Increased outdoor biting tendency of *Anopheles arabiensis* and its challenge for malaria control in Central Ethiopia. Public Health.

[52] Killeen GF, Govella NJ, Lwetoijera DW, Okumu FO (2016). Most outdoor malaria transmission by behaviourally resistant *Anopheles arabiensis* is mediated by mosquitoes that have previously been inside houses. Malar J.

[53] Ogola EO, Fillinger U, Ondiba IM, Villinger J, Masiga DK, Torto B (2018). Insights into malaria transmission among *Anopheles funestus* mosquitoes. Kenya Parasit Vectors.

[54] Matowo NS, Martin J, Kulkarni MA, Mosha JF, Lukole E, Isaya G (2021). An increasing role of pyrethroid-resistant *Anopheles funestus* in malaria transmission in the Lake Zone, Tanzania. Sci Rep.

[55] Wanjala CL, Kweka EJ. Malaria vectors insecticides resistance in different agroecosystems in Western Kenya. Front Public Health. 2018;6. 10.3389/fpubh.2018.00055 PMC5838019 29546039

[56] Machani MG, Ochomo E, Amimo F, Mukabana WR, Githeko AK, Yan G (2022). Behavioral responses of pyrethroid resistant and susceptible *Anopheles gambiae* mosquitoes to insecticide treated bed net. PLoS ONE.

[57] Sherrard-Smith E, Skarp JE, Beale AD, Fornadel C, Norris LC, Moore SJ (2019). Mosquito feeding behavior and how it influences residual malaria transmission across Africa. Proc Natl Acad Sci.

[58] Walch OJ, Cochran A, Forger DB (2016). A global quantification of “normal” sleep schedules using smartphone data. Sci Adv.

[59] Beier JC, Wilke AB, Benelli G. Newer approaches for malaria vector control and challenges of outdoor transmission. Towards malaria elimination—a leap forward; 2018.

